# Sustained Complete Molecular Remission With Imatinib Monotherapy in a Child Presenting With Blast Phase *FIP1L1-PDGFRA*-Associated Myeloid Neoplasm With Eosinophilia

**DOI:** 10.1097/HS9.0000000000000486

**Published:** 2020-11-06

**Authors:** Juhi Jain, Elizabeth P. Weinzierl, Debra Saxe, John Bergsagel, Jason Gotlib, Andreas Reiter, Sunil S. Raikar

**Affiliations:** 1 Department of Pediatrics, Aflac Cancer and Blood Disorders Center, Children's Healthcare of Atlanta and Emory University, Atlanta, Georgia, USA; 2 Department of Pathology and Laboratory Medicine, Children's Healthcare of Atlanta, Atlanta, Georgia, USA; 3 Department of Pathology and Laboratory Medicine, Emory University, Atlanta, Georgia, USA; 4 Department of Medicine, Stanford Cancer Institute, Stanford University, Stanford, California, USA; 5 Department of Hematology and Oncology, University Hospital Mannheim, Mannheim, Germany.

The etiology of hypereosinophilia is divided into 2 categories, clonal or reactive in origin. The clonal form typically presents as a chronic myeloproliferative neoplasm (MPN), associated with rearrangements involving *PDGFRA/B*, *FGFR1* or with the *PCM1-JAK2* fusion.^1^ These fusion genes result in a constitutively active tyrosine kinase, and the *PDGFR*-rearrangements are very sensitive to tyrosine kinase inhibitors (TKIs) such as imatinib. More rarely, these rearrangements can be seen in cases of acute myeloid leukemia (AML), T-cell lymphoblastic leukemia/lymphoma (T-ALL/T-LLy), or mixed phenotype acute leukemia (MPAL), generally associated with eosinophilia.^1^ Given the varied presentation, the World Health Organization now classifies these malignancies under the category of “myeloid/lymphoid neoplasms with eosinophilia (MLN-Eo) and rearrangement of *PDGFRA*, *PDGFRB*, or *FGFR1*, or with *PCM1-JAK2*”.^1^ This disease is extremely uncommon in children and only 8 *FIP1L1-PDGRFA* cases have previously been described.^2–9^

A rare presentation of this entity, only previously reported in adults, is blast phase disease with >20% blasts in the peripheral blood (PB) or bone marrow (BM), and/or as extramedullary disease.^1,10,11^ Such a presentation is often difficult to distinguish from AML, and is typically accompanied with massive splenomegaly and peripheral eosinophilia. Interestingly, the adult literature has shown that blast phase cases with *PDGFR*-rearrangements respond remarkably well to imatinib monotherapy, with intensive chemotherapy rarely inducing durable remissions.^10,11^ Here, we report the first pediatric case of a myeloid neoplasm with eosinophilia associated with a *PDGFR-*rearrangement that presented in blast phase. The patient had an excellent response to low-dose imatinib monotherapy and remains in complete molecular remission 2 years after his initial diagnosis.

**Figure 1:**
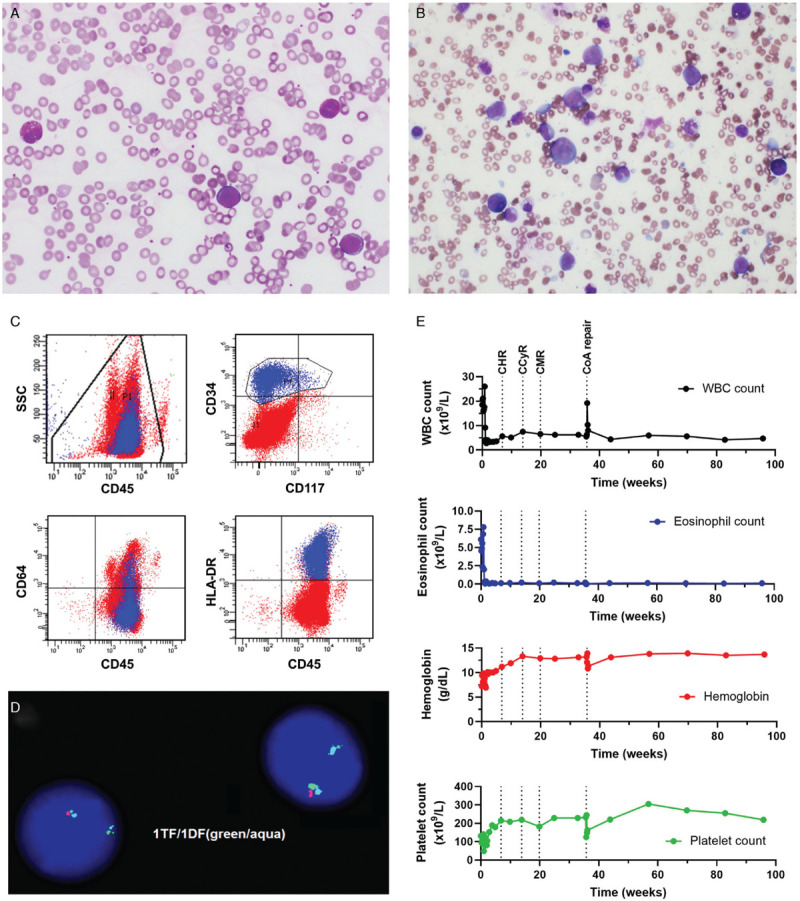
**Case features at presentation and treatment response to imatinib monotherapy.** (A) Peripheral blood smear shows a mild increase in white blood cells with a prominent blast population, morphologically enumerated at 11%. The blasts are intermediate in size with high nuclear to cytoplasmic ratios, fine chromatin, occasionally prominent nucleoli, and scant agranular basophilic cytoplasm. Increased eosinophils are also seen. (B) Bone marrow aspirate depicts a prominent population of blasts, which range from small to intermediate to large in size with high to moderate nuclear to cytoplasmic ratios, fine chromatin, occasionally prominent nucleoli, smooth nuclear contours, and basophilic cytoplasm. No Auer rods are seen. Blasts are enumerated at 23%. Some eosinophils demonstrate cytoplasmic vacuolization as well as occasional darker, basophilic granules. (C) Peripheral blood flow cytometry demonstrates a minimally differentiated expanded myeloid blast population (highlighted in blue) with a marked accompanying eosinophilia. The blasts overall express partial CD13, CD33, CD34 (shown), dim partial CD38, CD58, dim partial CD64 (shown), CD123, HLA-DR (shown), and dim CD45 (shown). CD117 (shown) and myeloperoxidase (MPO) are not expressed. (D) Fluorescence *in situ* hybridization (FISH) studies demonstrating the deletion of the *CHIC2* gene and fusion of *FIP1L1-PDGFRA* on 4q12. Normal pattern is two triple fusion (TF) signals (red, green and aqua). Cells that are positive for a *FIP1L1-PDGFRA* rearrangement show one double fusion (DF) signal (green and aqua only, deletion of *CHIC2* red), and a normal triple fusion on the normal chromosome 4. (E) Imatinib was started five days after initial presentation. A complete hematological response (CHR), complete cytogenetic response (CCyR) and complete molecular response (CMR) was attained 6, 13 and 19 weeks post initiation of treatment respectively. Trends in white blood cell (WBC) count (black), eosinophil count (blue), hemoglobin (red), and platelet count (green) is shown over the treatment period. Surgical repair of the patient's coarctation of aorta (CoA) was performed after 35 weeks of imatinib therapy.

Our patient, a 7-year-old male, presented with a 2-week history of fatigue, fever and abdominal distension for 2 days. Physical exam demonstrated massive splenomegaly. His complete blood count revealed a white blood cell count of 18.5 × 10^9^/L with 33% eosinophils (eosinophil count 6.1 × 10^9^/L) and 11% peripheral blasts (Fig. 1A), a hemoglobin of 7.2 g/dL and platelet count of 131 × 10^9^/L. Laboratory evaluation revealed an increased lactate dehydrogenase and uric acid, mild coagulopathy, and a significantly elevated vitamin B12 (>1400 ng/L) and tryptase level (14.4 ug/L). PB flow cytometry evaluation showed an abnormal myeloblast population expressing CD9 (partial), CD13 (dim partial), CD33, CD34, CD38 (dim partial), CD58, CD64 (dim partial), CD123, HLA-DR, and CD45 (dim). Myeloperoxidase and CD117 was not expressed (Fig. 1C). BM evaluation confirmed 23% abnormal myeloblasts with marked eosinophilia (Fig. 1B). The myeloblasts had a minimally differentiated AML immunophenotype, very different from the French-American-British (FAB) M4Eo AML subtype (acute myelomonocytic leukemia with eosinophilia), a common cause of eosinophilia-associated pediatric AML. Chromosomal analysis revealed twenty metaphases with a normal male karyotype 46,XY. Initial fluorescence in situ hybridization (FISH) analysis was negative for inv(16)(p13.1q22) or t(16;16)(p13.1;q22), rearrangements that cause the M4Eo AML *CBFB-MYH11* fusion. Apart from this, it was also negative for monosomy 5 or del(5q), monosomy 7 or del(7q), t(8;21), t(9;22), as well as negative for translocation or deletion of *KMT2A*. Given the significant hypereosinophilia, an extended FISH analysis was performed for *PDGFRA/B*, *FGFR1*, and *PCM1-JAK2* fusions. This confirmed the presence of a *CHIC2* deletion, a surrogate for the *FIP1L1-PDGFRA* fusion, in 60% of cells (Fig. 1D). The *FIP1L1-PDGFRA* fusion was also confirmed by nested reverse transcription polymerase chain reaction (RT-PCR). This established the diagnosis of “myeloid neoplasm with eosinophilia associated with *FIP1L1-PDGFRA*”. The bone marrow blast count of >20% was consistent with blast phase.

Based upon the adult experience in managing blast phase *FIP1L1-PDGFRA* patients,^10^ we chose to treat the patient with low-dose imatinib monotherapy at 100 mg daily (∼100 mg/m^2^/day) and avoided conventional high-dose cytotoxic chemotherapy. The patient had an impressive response to imatinib, with a rapid decline in blast count, eosinophilia and splenomegaly. The patient attained a complete hematologic remission (CHR), a complete cytogenetic remission (CCyR) and a complete molecular remission (CMR) after 6, 13, and 19 weeks of treatment respectively (Fig. 1E). His vitamin B12 and tryptase level reverted to normal. Interestingly at presentation, the patient was noted to have persistent hypertension, and further work-up revealed a previously undiagnosed coarctation of aorta with transverse aortic arch hypoplasia. After initial management with multiple anti-hypertensive medications, he underwent a successful surgical repair with an ascending aortic slide aortoplasty, nine months after his initial diagnosis. He is now off all anti-hypertensive medications. Our current management includes continuing imatinib with nested RT-RCR monitoring every 3 months. He has tolerated imatinib well with no change in cardiac function and normal growth velocity on the current dosing. He remains in complete molecular remission now two years post his initial diagnosis.

MLN-Eo can be best characterized as a pluripotent stem cell disorder driven by a constitutively active tyrosine kinase that exhibits both myeloid and lymphoid differentiation with sustained eosinophilia.^1^ Given its common presentation as a chronic MPN with eosinophilia, it was previously called chronic eosinophilic leukemia. However, recent literature has shown it can also present as AML, T-ALL/T-LLy or MPAL. Organ dysfunction of the skin, heart, lungs and digestive tract can occur due to eosinophilic infiltration. There are more than 70 fusion genes associated with MLN-Eo.^1^*PDGFRA* has eight known partner genes, with the most frequent being *FIP1L1*. The *FIP1L1*-*PDGFRA* fusion is caused by a cytogenetically cryptic submicroscopic 800-kb interstitial deletion on chromosome 4, del(4)(q12q12), containing the *CHIC2* gene.^1^ Most PDGFR-fusion proteins are very sensitive to the TKI imatinib. High rates of CHR and CMR are reported in adult studies with imatinib alone, with doses ranging from 100 to 400 mg/day.^1^ Among the eight pediatric *FIP1L1-PDGFRA* cases summarized in Table 1, seven presented as chronic MPNs with eosinophilia, while one presented as T-ALL/T-LLy without eosinophilia.^2–9^ Only 1/7 chronic MPN cases had elevated BM blasts at 10%.^8^

**Table 1:** Summary of Pediatric Cases with *FIP1L1-PDGFRA*-associated Myeloid/Lymphoid Neoplasm with Eosinophilia in the Literature.

Age (years)	Gender	Presenting features	Disease type	Initial WBC count (×10^9^/L)	Initial eosinophil count (×10^9^/L)	Treatment	Clinical course	Reference
6	Male	Pruritus, malaise	Chronic MPN, chronic phase (no reported BM blasts)	13.50	6.08	Prednisone	CHR at one month with prednisone. Relapsed after prednisone withdrawal, but re-attained remission once prednisone restarted. Plan was to start imatinib if patient relapsed again	Rives et al. 2005^2^
16	Male	Splenomegaly, lymphadenopathy, restrictive cardiomyopathy	Chronic MPN, chronic phase (no reported BM blasts)	13.50	5.28	Imatinib 400 mg daily	Attained CHR and CMR with imatinib, last follow-up at 36 months	Rapanotti et al. 2010^3^
14	Male	Pallor, weight loss, left shoulder pain, hepatosplenomegaly, inguinal lymphadenopathy	Chronic MPN, chronic phase (<5% BM blasts)	131.09	49.00	Hydroxyurea, then imatinib 100 mg bid	CHR at 14 days, CCyR at 110 days, CMR at 230 days	Farruggia et al 2014^4^
2	Female	Malaise, fatigue, loss of appetite, pain	Chronic MPN, chronic phase (no reported BM blasts)	125.00	22.50	Imatinib 300 mg/m2 daily	CHR at 2 weeks, CMR at 3 months. Discontinued imatinib treatment after 5 years. 5 months after discontinuation patient relapsed, restarted on 300 mg imatinib daily and after 4 weeks was back in remission	Rathe et al 2014^5^
9	Female	Malnutrition, fever, cough, diarrhea, cardiac dysfunction, lymphadenopathy, hepatosplenomegaly, polymorphous pruritic skin eruptions since one month of age, bilateral keratomalacia with corneal erosions at 3 years, stroke at 7 years.	Chronic MPN, chronic phase (no reported BM blasts)	Not reported	28.98	Steroids, imatinib 100 mg daily	CHR at 6 weeks, resolution of skin lesions, cardiac dysfunction	Zeng et al 2015^6^
13	Male	Right supraclavicular lymphadenopathy, no eosinophilia	T-cell lymphoblastic leukemia/ lymphoma	1.40	0	Standard chemotherapy, imatinib added at relapse	Refractory disease with progression after 109 days. FiP1L1-PDGFRA fusion detected on re-evaluation, imatinib added to chemotherapy. Minimal response, had subsequent progression leading to death.	Oberley et al 2017^7^
15	Male	Migrating joint pain, splenomegaly	Chronic MPN, accelerated phase (10% BM blasts)	49.16	16.71	Hydroxyurea, then imatinib 100 mg daily. Switched to 200 mg weekly after 18 months	CHR at one month, CCyR at 3 months. Remained in remission on maintenance imatinib ∼42 months from diagnosis	Srinivasan et al 2019^8^
5	Female	Multifocal bone pain, fever, fatigue, malaise, anorexia, weight loss, vomiting, bloody diarrhea, headaches, cardiomyopathy, hepatosplenomegaly, lymphadenopathy	Chronic MPN, chronic phase (no reported BM blasts)	59.97	29.43	Systemic corticosteroids for 2 weeks, imatinib 100 mg daily. Reduced to 100 mg every other day at 9 months	CMR at 9 months, reduced imatinib dosing. Remained in remission for 16 months thereafter at last follow-up, cardiomyopathy completely resolved.	Bota et al 2019^9^
6	Male	Fever, fatigue, splenomegaly	Chronic MPN, blast phase (23% BM blasts)	18.50	6.11	Imatinib 100 mg daily	CHR at 6 weeks, CCyR at 13 weeks, CMR at 19 weeks. Remains in remission 2 years since diagnosis	Current case

BM = bone marrow, CCyR = complete cytogenetic remission, CHR = complete hematologic remission, CMR = complete molecular remission, MPN = myeloproliferative neoplasm, WBC = white blood cell.

This disease rarely presents in blast phase, defined by >20% PB/BM blasts and/or extramedullary disease.^1,10,11^ To date, this has only been reported in adults, with our case being the first instance of a child presenting with blast phase *FIP1L1-PDGFRA* disease. This is a challenging diagnosis to make, and can easily be mistaken for AML if the clinical suspicion is not high for a clonal eosinophilic process. The more common eosinophilia-associated myeloid neoplasm in children is the FAB M4Eo AML subtype associated with the *CBFB-MYH11* fusion, which is routinely tested for in de novo AML.^1^ Therefore, all *CBFB-MYH11-*negative myeloid neoplasms with eosinophilia should warrant additional screening for *PDGFR*, *FGFR1,* and *PCM1-JAK2* fusions. Making the accurate diagnosis is critical, especially in cases associated with *PDGFR*-translocations, as it greatly affects the overall treatment approach.

Blast phase *BCR-ABL1*-positive chronic myeloid leukemia (CML) is typically treated with a TKI plus intensive chemotherapy followed by allogeneic hematopoietic stem cell transplantation (HSCT)^12^; however, the approach to treating blast phase *PDGRF*-rearranged MLN-Eo is vastly different. Adult studies have shown that sustained remission is achievable with imatinib alone, without high-dose chemotherapy or allogeneic HSCT.^10,11^ In a study of 17 adults with *PDGFRA/B* fusion-positive blast phase (myeloid/lymphoid) or chloromas, the 15 patients treated with imatinib monotherapy achieved CHR.^10^ CMR was attained in all 12 *FIP1L1-PDGFRA-*positive patients, at a median of five months. One *FIP1L1-PDGFRA* patient underwent allogeneic HSCT after failing to achieve remission with chemotherapy. Unfortunately, the patient relapsed early, but later on imatinib alone had a sustained CMR for 9 months post-HSCT.^10^ Another study with five *PDGFRB* blast phase patients showed that all three patients who underwent allogeneic HSCT relapsed.^11^

Cardiac injury in adults with *FIP1L1-PDGFRA*-related disease is a multi-step process initiated by eosinophilic damage to the endocardium manifesting as cardiomyopathy, constrictive pericarditis, endomyocarditis, valvular dysfunction, and myocardial infarction.^13^ To our knowledge, no *FIP1L1-PDGFRA* case has involved coarctation of aorta. It remains unclear in our case if there is a correlation between the genetic abnormality and his cardiac disease. While the decision to treat with imatinib monotherapy was not influenced by the discovery of a concurrent cardiac abnormality, it allowed for surgical correction that would have been difficult in the setting of intensive chemotherapy. Furthermore, the prompt resolution of eosinophilia prevented any cardiac dysfunction, which could have been life threatening given the concurrent aortic coarctation.

Given the excellent response to imatinib observed in our patient, our plan is to continue imatinib monotherapy indefinitely and not pursue allogeneic HSCT. Resistance to imatinib in *FIP1L1-PDGFRA*-related disease can occur due to the T674I mutation within the ATP-binding domain of *PDGFRA*, which is analogous to the *BCR-ABL1* T315I mutation in CML, and confers pan-resistance to multiple TKIs except ponatinib.^1,14^ Another cause is the D842 V mutation in the activation loop of *PDGFRA*, which can be targeted by ponatinib or avapritinib.^1,14^ If resistance were to develop in our patient, we would use ponatinib to achieve a second remission followed by allogenic HSCT. There is no clear evidence in the pediatric literature as to when to stop therapy in these cases. Among the pediatric cases, one patient with chronic phase *FIP1L1-PDGFRA* disease was taken off imatinib after 5 years of sustained molecular remission, but subsequently relapsed within 5 months.^5^ After re-starting imatinib, the patient was able to achieve remission again. Thus, our patient will most likely require lifelong therapy. Close monitoring for late effects of imatinib is required, especially in terms of growth velocity, which is known to be affected in pediatric *BCR-ABL1*-positive cases receiving long-term TKI therapy.^15^
